# Are the Processes Underlying Discrimination the Same for Women and Men? A Critical Review of Congruity Models of Gender Discrimination

**DOI:** 10.3389/fpsyg.2019.00469

**Published:** 2019-03-06

**Authors:** Francesca Manzi

**Affiliations:** Department of Psychology, New York University, New York, NY, United States

**Keywords:** gender stereotypes, role congruity theory, lack of fit, gender discrimination, male targets

## Abstract

Although classic congruity models of gender discrimination (e.g., role congruity theory, lack of fit) predict negative outcomes for both women and men in gender-incongruent domains, the literature has focused almost exclusively on discrimination against women. A number of recent studies have begun to address the question of whether and under what circumstances men can also be the targets of gender discrimination. However, the results of these studies have so far been mixed. Therefore, the question of whether men, like women, also suffer discrimination when in gender incongruent roles and domains remains unclear. The goal of the present paper is to integrate and critically examine the burgeoning literature on gender discrimination against men in order to assess whether the symmetrical predictions of congruity models are supported. Through this close analysis and integration of the literature, I aim to identify remaining gaps in the research on gender discrimination. In particular, I propose that researchers of gender discrimination would benefit from expanding their scope beyond that of paid work.

## Introduction

At first glance, research in the social sciences appears to have provided a thorough account of the dynamics underlying gender-based discrimination. Social psychology in particular has produced a large literature that has sought to uncover the cognitive and motivational mechanisms behind gender discrimination, as well as to track changes in the nature of gender discrimination over time. However, the majority of research in gender discrimination has focused almost exclusively on discrimination against women in traditionally male roles and occupations (Jetten et al., 2013).

This focus on women has not been arbitrary—discrimination on the basis of gender has been a particular problem for women, especially in employment settings. Further, even though women now comprise nearly half of the workforce in most developed nations (Pew Research Center, 2017c; United States Bureau of Labor Statistics, 2017), there are still important domain-specific gender imbalances, such that women remain dramatically underrepresented in occupations that have been traditionally dominated by men. This imbalance puts women at an important social and economic disadvantage, as these positions tend to hold the highest prestige and status, as well as higher monetary and social rewards (Cejka and Eagly, 1999; Hegewisch and Hartmann, 2014; Levanon and Grusky, 2016). Because gender-based discrimination has historically interfered with women’s professional success and continues to hinder their social mobility, gender bias against women is an obvious and central impediment to gender equality. Thus, the focus on gender discrimination against women – and not men – makes sense from a historical, cultural, and political point of view.

The fact that discrimination continues to affect women more than men, however, does not necessarily mean that men cannot be the targets of gender bias in evaluation. Although empirical research has focused almost exclusively on women, most psychological theories of the antecedents and consequences of gender discrimination are not meant to be gender-specific. Rather, many of these theories are posited as explanations of gender bias more generally and therefore should also be able to account for patterns of discrimination against men, should they exist. Though these social psychological theories about gender discrimination have shown themselves to be useful in explaining why, when, and how women encounter barriers in traditionally male roles and occupations, whether they can also explain the potential limitations men encounter when seeking entry into traditionally female domains remains to be seen. Thus, examining whether and under which circumstances men are discriminated against on the basis of their gender has important theoretical implications.

The goal of the present paper is to critically examine classic models of gender discrimination by expanding their scope beyond women in traditionally male settings to also integrate research on the evaluation of men in traditionally female roles and occupations. The primary focus of this review is on congruity models of discrimination (hereafter, “CMDs”) such as “role congruity theory” (Eagly and Karau, 2002), “lack of fit” (Heilman, 1983, 2012), and “think manager, think male” (Schein, 1973, 2001), which are among the most well-examined and empirically supported theories of gender bias in the psychology literature. These theoretical explanations argue that there can be a mismatch between what men and women are perceived to be like (i.e., gender stereotypes) and what is thought to predict success in specific occupations (i.e., job stereotypes). This perceived mismatch or incongruity between gender stereotypes and job stereotypes leads to negative performance expectations for both women and men in gender-incongruent domains and, in turn, gives rise to gender discrimination.

The predictions made by CMDs have been consistently supported in research on bias against women in stereotypically masculine (i.e., “male-typed”) settings. However, the accuracy of these theories in predicting whether men face similar biases in stereotypically feminine (i.e., “female-typed”) occupations and roles is less well established. Accordingly, the primary goal of this paper is to review the existing literature in order to examine whether the processes affecting discrimination against men and women are symmetrical (i.e., whether being in a gender-incongruent role has similar negative effects for both men and women). In doing so, this review will assess the core tenets of CMDs and the psychological mechanisms that they contend are responsible for giving rise to gender discrimination.

Exploring whether men can be the targets of gender-based bias is important not only from a theoretical perspective, but also from a practical one. While women’s entry and participation in traditionally male domains have increased dramatically in the past decades, men’s participation in traditionally female domains has remained stubbornly stagnant (Blau et al., 2013). Given that occupations in which women outnumber men are typically devalued (Cohen and Huffman, 2003; Hegewisch and Hartmann, 2014), increasing male participation in these areas may help decrease gender segregation and, in turn, help balance the prestige and economic rewards that are allocated to both male- and female-dominated occupations. Importantly, if men’s under-representation in feminine roles can be explained, even in part, by traditional models of gender discrimination, then the knowledge we have gained from decades of research on women in traditionally male-settings should be helpful in identifying strategies to combat anti-male bias. If, on the other hand, men’s lack of participation in female roles and occupations is not due to gender discrimination, or if the processes underlying bias are not analogous for women and men, then there may be a need for both theoretical revision, as well as new ways to address the persistent gender imbalance in the workplace.

In the following sections, I will review the extant literature focusing on the evaluation of men in female-dominated occupations and interpret these results in light of the predictions made by CMDs. In keeping with the dominant approach of research on CMDs, this review will focus primarily on the processes underlying gender discrimination from the evaluator’s perspective (rather than that of the “target” or person being evaluated). That is, the focus will be on people’s judgments and evaluations of *other* men’s and women’s occupational competence. Because the predictions of CMDs center on evaluations of men in female-typed domains, this review will also be limited to perceptions of men in female-typed roles and occupations.

## Can Men Be the Targets of Gender Discrimination?

In recent years, there has been a rise in perceptions of anti-male discrimination. Over 40% of adults believe that men face a little or a moderate amount of discrimination in the United States (American National Election Studies, 2016). While the percentage of men alleging that they have suffered some form of discrimination on account of their gender is still far below that of women (22% vs. 42%, respectively, Pew Research Center, 2017b), many men believe that anti-male discrimination is on the rise, and that it is more prevalent today than in past decades (Bosson et al., 2012; Kehn and Ruthig, 2013).

What explains these growing perceptions of anti-male discrimination? They may in part be a consequence of women’s recent social advancements and the appearance of gender-related initiatives focused on women. For example, some see the increase in academic diversity programs aimed at girls, but not boys, as discriminatory, especially given that women are now more highly educated than men (Coston and Kimmel, 2012; Okahana and Zhou, 2018). Diversity policies such as affirmative action or gender quotas may also be seen as discriminatory because they are thought to violate meritocracy (Eberhardt and Fiske, 1994).

However, perceptions of discrimination against men can also be motivated in nature. For example, believing that diversity policies are based on unjust processes has been found to protect men’s self-esteem when confronted with negative performance feedback (Unzueta et al., 2008). Furthermore, men’s perception that they too are victims of discrimination may be a form of competitive victimization. According to this perspective, claiming victimhood is a reaction to men’s dominance being threatened and/or to feelings of guilt about men’s higher social standing (Kobrynowicz and Branscombe, 1997; Sullivan et al., 2012; Jetten et al., 2013; Dover et al., 2016; Young and Sullivan, 2016).

Although the belief that men experience discrimination has been on the rise among the general public, this idea has been far more contentious in academic research and theory. Some argue that, because of their social standing, men are less threatened than women by gender-based bias because gender discrimination does not impede men’s upward mobility (Jetten et al., 2013). However, although men may suffer fewer negative outcomes as a result of discrimination, this does not mean that discrimination against men cannot occur. Gender-based discrimination is generally defined as any behavior or action that results in the unfavorable treatment of a person because of their sex or gender (Heilman and Manzi, 2016), and past work has suggested that, under certain circumstances, men too can be subject to negative treatment because of the gender group to which they belong (e.g., Heilman and Wallen, 2010; Vandello and Bosson, 2013). Thus, although the nature and consequences of discrimination may be very different for women and men, men can also be the targets of gender discrimination, at least by this definition.

Nevertheless, other theoretical perspectives contend that definitions of discrimination should also incorporate the notion of legitimacy. Such perspectives are reflected in many mainstream psychological definitions of prejudice, which stipulate that the negative treatment of group members must be “unfair” or “unjustified” in order to constitute discrimination (Major et al., 2002). One potential problem with this definition lies in the fact that what is perceived to be justified or unjustified can vary greatly as a function of many factors, such as changing societal norms. It is likely that actions and circumstances that most people now unequivocally categorize as discrimination against women were not always perceived as such. Under contemporary standards, it is difficult to imagine that, less than 100 years ago, women were not allowed to vote in most countries. Or that up until the 1970s, United States companies could legally terminate pregnant women if they saw them as a liability for their business (United States Equal Employment Opportunity Commission, 1978). Even until recently, non-consensual sex within marriage was not considered rape in most countries (and remains decriminalized in many; World Bank, 2015).

In the modern day, the subjective nature of perceptions of what is “just” is illustrated by other societal practices that appear to go unquestioned. For example, it is still common for wives to take their husband’s last name after marriage in many western countries (e.g., Gooding and Kreider, 2010). The reverse practice – the husband adopting the wife’s surname – is rare, and the legal procedures that a newly wed couple must go through to make this arrangement are often more obtuse than taking the traditional route (Rosensaft, 2002; Weisberg and Appleton, 2015). Despite this imbalance, most US men and women consider this practice to be perfectly acceptable and more than half believe that women should be required to adopt their husband’s last name (Hamilton et al., 2011).

Cultural norms that continue to proscribe traditionally feminine behavior for men may also play a role in why discrimination against men is often not labeled as such. For example, people may be more accepting of behaviors that serve to reinforce these norms, such as challenging the masculinity of male nurses, questioning the competence of a male nanny, or derogating men who actively seek out family-friendly work opportunities (see Funk and Werhun, 2011; Vandello et al., 2013). In this way, social norms may grant a degree of legitimacy to actions that would otherwise be judged as unjust and harmful to men, preventing people from viewing them as discriminatory.

But detecting discrimination is not only a function of an action’s perceived legitimacy. Whether people judge an action to be discriminatory also depends on the actors involved – particularly who the perpetrator and the victim are. When the perpetrator is atypical, detecting discrimination becomes more difficult. For example, behavior is less likely to be perceived as discriminatory when it involves a less powerful group acting against a more powerful group (Baron et al., 1991; Inman et al., 1998; Barreto and Ellemers, 2005; Barreto et al., 2010). The perceived typicality of the target also shapes people’s judgments of whether discrimination has occurred. Victims of discrimination are less likely to be perceived as such if they do not belong to a group that is commonly discriminated against – that is, when they are not prototypical victims (Inman and Baron, 1996). As members of a high-status group, men are atypical targets of discrimination. Thus, even if there are circumstances in which men are treated negatively because of their gender, they may be less likely to be perceived as victims of gender-based bias.

## Can Discrimination Occur in Female-Typed Roles and Occupations?

In addition to a focus on women as the targets of discrimination, gender research has also overwhelmingly focused on discrimination in male-typed contexts – that is, occupations that have been historically dominated by men and/or are thought to require stereotypically masculine characteristics. Male-typed occupations typically hold more power and prestige, so it is not surprising that researchers would direct their efforts toward identifying the barriers to women’s access and advancement within this domain. However, this narrow focus has left social psychology little insight into the forces at work in female-typed domains such as early childhood education, health care, and domestic labor.

The lack of research on discrimination in female-typed domains may also reflect the fact that being restricted from entering these domains may often not be categorized as discrimination. Compared to male-typed occupations, traditionally female occupations are generally devalued, tending to carry less status and monetary rewards (England, 2010; Blau and Kahn, 2017). As a result, being excluded from these occupations on the basis of gender may not be readily seen as discrimination, as the social and economic consequences of this exclusion may be less evident.

Importantly, the interplay between the status of the target of discrimination and the status of the occupation may also have implications for whether people perceive an event as discriminatory. Specifically, the lower status of women (relative to men) and the higher status of male-typed occupations (relative to female-typed occupations) are seen to represent an upward movement for women in these fields. Therefore, biases that limit women’s full access to these occupations often result in women’s relegation to a lower-status position, serving to curb their progress and upward mobility. Conversely, the consequences for men in female-typed occupations are less straightforward. Participation in areas that have been historically dominated by women may be seen to represent a downward movement for men. Thus, actions that result in men’s exclusion from female-typed occupations may appear to others as less egregious. Research shows that an event is more likely to be perceived as discriminatory when it is thought to cause significant harm to the victim (Swim et al., 2003). Therefore, even if men are excluded from female domains because of their gender, and even if the processes underlying this exclusion are similar to those suffered by women in traditionally male domains, such an event may not be deemed discriminatory because it is not seen as particularly harmful for men. Furthermore, because the consequences of discrimination against men in these settings are also less prototypical, people may be less prone to recognizing gender-based discrimination within settings historically dominated by women. In this way, the differential social and economic value assigned to traditionally female versus male activities may be playing an important role in shaping whether, when, and where discrimination is perceived to take place.

In sum, the consequences of discrimination against women and men likely differ in many ways, and men’s historical advantage and higher social status may to some degree shield them from some of the negative outcomes that women often experience. Moreover, the domains in which gender discrimination against men should be most likely to occur – female-typed roles and occupations, according to CMDs – are typically devalued. Likely as a result of this non-prototypical scenario, discrimination against men in traditionally female domains has often not been labeled as such. Nevertheless, men, like women, can suffer negative outcomes as a function of the specific gender group to which they belong. When and why men experience these negative outcomes has important theoretical implications for our understanding of gender-based bias.

## Congruity Models of Gender Discrimination

A large body of work exploring the mechanisms underlying gender-based discrimination has shown that women’s and men’s participation in the workplace is affected by gender bias in evaluation – a bias that has its origins in gender stereotypes (Burgess and Borgida, 1999; Cejka and Eagly, 1999; Eagly and Karau, 2002; Heilman, 2012). Gender stereotypes are shared beliefs about the attributes, personality traits, and abilities of women and men. Regardless of their (real or perceived) accuracy, gender stereotypes affect how we perceive and evaluate others (Bussey and Bandura, 1999; Eagly and Wood, 2013; Ellemers, 2018). In this way, gender stereotypes often lead to discrimination by guiding decision-making processes in the direction of stereotype-consistency.

Gender stereotypes are developed and perpetuated through the differential distribution of roles and occupations in society. Men’s overrepresentation in breadwinning roles and high-power occupations has led to stereotypes portraying men as particularly agentic. Similarly, women’s overrepresentation in domestic roles and caregiving occupations has aligned female stereotypes with communality (Eagly et al., 2000; Koenig and Eagly, 2014). Agency comprises attributes such as achievement orientation (e.g., able, successful), assertiveness (e.g., dominant, forceful), and autonomy (e.g., independent, self-reliant), while communality denotes consideration for others (e.g., caring, helpful), affiliation with others (e.g., sociable, likable), and emotional sensitivity (e.g., tender, sensitive). Continuous exposure to this gendered division of labor also gives rise to the belief that men and women are fundamentally different. That is, men are thought to be more agentic than communal, and women are thought to be more communal than agentic (Broverman et al., 1972; Kite et al., 2008; Wood and Eagly, 2010).

Importantly, gender stereotypes are both descriptive and prescriptive. That is, they depict what men and women *are* like as well as what men and women *should be* like. The descriptive component of gender stereotypes comprises beliefs about the characteristics of each gender group (e.g., women are emotional, men are rational), while the prescriptive component establishes norms about the appropriate behavior of men and women (e.g., women should be caring, men should be strong) (Burgess and Borgida, 1999; Prentice and Carranza, 2002). Both descriptive and prescriptive components of gender stereotypes have implications for the differential recruitment, selection, and promotion of men and women into different occupations. However, the processes by which descriptive and prescriptive stereotypes give rise to gender-based discrimination vary. Namely, descriptive stereotypes lead to discrimination through differential perceptions of male and female competence in specific roles and occupations, and prescriptive stereotypes lead to discrimination through the derogation and social penalization of male and female norm-violators (Heilman, 2012; Rudman et al., 2012). Although prescriptive gender stereotypes undoubtedly contribute to the underrepresentation of both women and men in gender-incongruent domains, the focus of the review that follows will primarily be on descriptive gender stereotypes, as these have been the main focus of CMDs.

### The Effect of Descriptive Stereotypes: Discrimination as Perceived Incompetence

According to CMDs, descriptive stereotypes depicting women as communal and men as agentic do not always lead to negative outcomes. Rather, gender discrimination arises when these stereotypes conflict with what is thought to predict success in specific roles and occupations. Though different jobs certainly require different competencies for successful performance (e.g., being a good nurse requires more biology knowledge than being a good journalist), their *perceived* requirements and the relative importance ascribed to each are also informed by gender stereotypes. For example, jobs in which women are heavily overrepresented (e.g., bank tellers, dental hygienists) tend to be seen as requiring more communal characteristics than occupations in which men are the majority (e.g., financial adviser, civil engineers), which are seen as requiring more agentic characteristics (Glick et al., 1995; Cejka and Eagly, 1999). In this way, occupations themselves are gendered, with the workplace largely being divided into “women’s work” and “men’s work” (Reskin and Hartmann, 1986; Ridgeway, 2011). Beliefs about the gender-type of different roles and occupations emerge very early (Liben et al., 2001; Martin and Ruble, 2004). Moreover the associations between men, women, and specific occupations (e.g., woman-nurse, man-surgeon) are automatic and difficult to suppress (Oakhill et al., 2005).

Congruity models of discrimination focus on this interplay between descriptive gender stereotypes and the gender-type of particular roles and occupations, arguing that gender-based discrimination is the result of a perceived mismatch between what men and women are thought to be like (i.e., agentic and communal, respectively) and the traits deemed necessary for job success. This perceived mismatch, in turn, gives rise to negative expectations about the potential for success of an individual in a gender-incongruent domain. That is, descriptive gender stereotypes lead to the belief that women and men are not well-equipped to perform effectively in occupations that have been historically dominated by the opposite sex and that they will therefore be less competent in these roles.

Theoretically, CMDs are “gender-blind.” They predict that discrimination occurs because of a perceived incongruity between female *or* male stereotypes and occupational stereotypes. Thus, CMDs predict a symmetrical effect: women will be deemed less competent than men in traditionally male domains, and men will be deemed less competent than women in traditionally female domains. In both cases, the outcome of these stereotype-based expectations should be gender discrimination.

## Gender Stereotypes and Bias in the Evaluation of Women

Congruity models of discrimination have been widely and successfully used to describe and predict anti-female bias in such disparate male-typed settings as the military (e.g., Boldry et al., 2001), upper-level management (e.g., Eagly and Carli, 2007), academia (e.g., Schmader et al., 2007), and sports (e.g., Koivula, 2001). Moreover, a large body of research has provided support for the predictions made by CMDs regarding the psychological mechanisms behind gender bias, with numerous studies demonstrating that stereotype-based expectations lead to discrimination at various stages of women’s lives and careers.

Anti-female bias in traditionally male domains begins early on. Female students are perceived as less intelligent and capable than their male peers in domains such as technology and science (Cheryan et al., 2017). This bias is also seen in parents, who often encourage their daughters to pursue more gender-congruent activities, thereby reinforcing beliefs about their lesser competence in male-typed domains (Leaper and Gleason, 1996; Tenenbaum and Leaper, 2003). Even when actively exposing their children to science (an area generally perceived as male in gender-type), parents dedicate more time and effort explaining scientific processes to their sons than to their daughters (Crowley et al., 2001). Gender-based bias continues in higher education, where women are perceived to be less talented than men in academic fields such as engineering, science and philosophy (Nosek et al., 2009; Moss-Racusin et al., 2012; Leslie et al., 2015).

For women who nonetheless choose to pursue traditionally male jobs, the mismatch between occupational stereotypes and female stereotypes gives rise to negative outcomes throughout their careers. Anti-female bias has been observed in job recruitment (e.g., Gaucher et al., 2011), in screening of application materials (e.g., Schmader et al., 2007), in selection decisions (e.g., Bosak and Sczesny, 2011), and in promotion opportunities (e.g., Lyness and Heilman, 2006; Hoobler et al., 2009). The existence of bias against women in male-typed jobs has received further support from several meta-analyses. A recent meta-analysis by Koch et al. (2015) provided strong support for the predictions made by CMDs for women in male-typed domains. In their analysis of 136 experimental studies, the authors found that women were evaluated less positively than men when the job was male in gender-type. In contrast with previous meta-analyses (e.g., Davison and Burke, 2000), the authors found that these effects were driven by male, but not female evaluators. Given that decision-makers in these occupations are likely to be men, these findings suggest that women in male-typed jobs continue to be highly vulnerable to gender-based discrimination.

In keeping with the predictions of CMDs, there is also evidence that the degree of bias against women in a specific male-typed occupation can also change if the stereotypes regarding what is necessary for that occupation change – supporting the contention that gender bias stems from a perceived mismatch between occupational stereotypes and stereotypes about women. Research suggests that such a change may be occurring in the domain of leadership. Early research found that stereotypes about leaders generally resembled stereotypes about men, creating the perception that men are more naturally equipped to fulfill these roles and leading to subsequent discrimination against women in a variety of leadership contexts (see Eagly et al., 1995). In the intervening decades, however, stereotypes about leaders appear to have incorporated more communal characteristics and discrimination against women in leadership roles seems to be decreasing (e.g., Sczesny et al., 2004; Koenig et al., 2011). In line with this change, a recent meta-analysis by Paustian-Underdahl et al. (2014) found no evidence of gender bias in people’s evaluations of female leaders in male-typed settings.

Providing further support for CMDs, other information that reduces incongruity perceptions has also been shown to reduce gender bias. Such an effect has been documented for individual women who are depicted as clearly counterstereotypical. For example, presenting an individual woman as unequivocally or exceptionally competent reliably reduces the gender bias against that woman in male-typed settings (Koch et al., 2015). Under certain circumstances, these strongly counterstereotypical women can even be preferred over men as they are often perceived to be *extraordinarily* competent (Correll and Ridgeway, 2006). Indeed, recent studies suggest that unambiguously successful women are favored over equally qualified men, even in highly male-typed domains (Williams and Ceci, 2015; Leslie et al., 2017). Thus, presenting an individual woman as a clear “exception to the rule” can reduce her perceived incongruity for a given role and, as a result, discrimination is greatly attenuated (or may even be reversed in her favor).

## Gender Stereotypes and Bias in the Evaluation of Men

In much the same way that success in male-dominated jobs is associated with agency, communality is perceived to be a requisite for success in traditionally female roles and occupations. Research supports this idea, demonstrating that female-dominated occupations and fields are more strongly associated with traditionally female than male traits (Cejka and Eagly, 1999; Gilbert et al., 2015). CMDs predict that the mismatch between people’s perceptions of female-typed occupations and male stereotypes will lead to the belief that men will be less competent than women in these settings. These negative competence expectations should, in turn, lead to anti-male bias and discrimination against men in traditionally female domains.

Although there is general consensus among gender researchers regarding women’s lower perceived competence in male-typed roles and occupations, there seems to be much less agreement about the consequences that men face when they find themselves in female-typed occupations. While some studies have provided support for CMDs by documenting anti-male bias in female-typed domains, others suggest that, far from suffering discrimination, men are actually favored over women in traditionally female occupations.

As discussed above, the extant research on evaluations of men in female-typed occupations is both scant and fairly recent. However, when considered together, many of these studies offer indirect support for CMDs. For example, men desert female-dominated college majors and occupations at significantly higher rates than women (Addi-Raccah, 2005; Stott, 2007; McLaughlin et al., 2010; Riegle-Crumb et al., 2016). This phenomenon appears to be analogous to what has been termed the “leaky pipeline,” referring to the comparatively higher rate of female attrition in male-typed domains (e.g., Cheryan et al., 2017; Department of Commerce of the United States of America, 2017). Thus, research suggests that similar trajectories and career development outcomes might exist for both men and women who choose to pursue gender-incongruent careers.

One possible explanation for these patterns is that the perceived incongruity between gender and occupation leads to higher attrition rates, as CMDs would predict. Supporting these predictions, there is evidence that men who leave female-typed domains are more likely to move into gender-balanced and male-dominated careers, even when this move results in a pay cut (Barnett et al., 2000; Addi-Raccah, 2005; Riegle-Crumb et al., 2016; Torre, 2018). It has been argued that this leaky pipeline may be due, at least in part, to a general culture within these domains that signals to men that they do not fit (O’Lynn, 2004; Simpson, 2004; Kermode, 2006; Bartfay et al., 2010; Isacco and Morse, 2015). Congruity beliefs can affect self-perceptions, which, in turn, may lead to negative outcomes for men in female-typed jobs. For example, perceiving greater conflict between their gender and their job has been linked to higher rates of depression and anxiety, as well as lower job satisfaction and commitment, among male nurses, early childhood educators, and flight attendants (Young and James, 2001; Wolfram et al., 2009; Wallen et al., 2014).

Taken together, these studies suggest that some form gender bias against men may exist in traditionally female fields. However, it is unclear whether and to what degree discrimination *per se* contributes to these negative outcomes, or whether they are due entirely to men’s own perceptions that they do not fit (see Schmader and Sedikides, 2018). That is, men may be deemed competent by others but still choose to leave female-dominated environments because they do not feel like they fully belong. Nevertheless, such perceptions are rarely formed “in a vacuum,” and it seems likely that there may be structural or interpersonal factors that contribute to men’s feelings of lack of fit.

Other research has provided more direct evidence in support of CMDs, suggesting that the mismatch between male stereotypes and the perceived requirements for success in female-typed domains leads to the expectation that men will not perform as well as women. Several qualitative studies suggest that men are seen as lacking the female skills considered necessary to be a good nurse, early educator, or caregiver (Hochschild, 1983; Yang et al., 2004; Bartfay et al., 2010; Hedlin and Åberg, 2013; Warming, 2013). Providing support for the role of gender stereotypes in this process, there is evidence that these expectations of male incompetence can give rise to stereotype threat among men in female fields. Mirroring the findings from a large body of research demonstrating that stereotype threat can affect women’s performance in male-typed tasks and occupations (Steele and Aronson, 1995; for a meta-analysis see Nguyen and Ryan, 2008), men’s performance in female-typed jobs and tasks is also impaired when stereotypes about women’s greater ability are made salient (Leyens et al., 2000; Koenig and Eagly, 2005; Kalokerinos et al., 2017).

Still, a central contention of CMDs is that the mismatch between gender stereotypes and the perceived requirements for success in gendered occupations should lead not only to expectations of incompetence, but also to discrimination. Thus, if men are deemed less competent in female-typed roles and occupations, there should be evidence of anti-male bias in selection processes, performance evaluations, and promotions. Some research provides support for this possibility, documenting more negative ratings of men than women applying for a traditionally female job (e.g., Cohen and Bunker, 1975; Gerdes and Kelman, 1981; Etaugh and Riley, 1983; Kim and Weseley, 2017). A recent audit study also revealed that, compared to equally qualified female applicants, male applicants received significantly fewer call-backs from employers in female-typed domains (Yavorsky, 2017). Moreover, a meta-analysis by Paustian-Underdahl et al. (2014) found a tendency for male leaders to be evaluated as less effective than female leaders in educational settings.

Taken together, this research provides some support for the idea that men too can be the targets of discrimination in female-dominated occupations. It also lends some support for the psychological mechanism posited by CMDs – that gender bias stems from presumptions of lesser male competence. However, the existing evidence is far from conclusive. Most of the studies reviewed above present rather indirect evidence for the symmetry predicted by CMDs, and very few show that there is a direct relationship between incongruity perceptions and anti-male discrimination in female-typed settings. These empirical gaps leave open questions regarding the processes underlying discrimination against men.

However, the greater challenge to CMDs may lie in the fact that there is a separate body of literature that appears to directly challenge the findings described above, suggesting that the exact opposite pattern of results can also occur. This research stems primarily from the work of Williams (1992, 1995b), who argued that not only do men not face discrimination in traditionally female jobs, they are actually preferred over women when applying for these jobs and tend to climb the organizational ladder more quickly. This male advantage in female-dominated jobs has been called the “glass escalator.” Williams (1992) argues that, unlike women in male-dominated settings, the gender of men in female-typed occupations is construed as a positive difference. As a result, male stereotypes work in men’s favor, helping rather than hindering their evaluations and upward mobility.

In line with this perspective, other research has suggested that the experience and consequences of underrepresentation are different for men than for women. For example, early research on “tokenism” contended that being an occupational minority (i.e., a “token”) heightens the visibility of one’s group membership. For women in male-typed jobs, this visibility leads to negative outcomes, as it makes gender stereotypes about women’s lesser competence in these fields salient to perceivers (e.g., Kanter, 1977; Crocker and McGraw, 1984). However, later work has shown that men actually benefit from their token status – the same visibility that leads to greater scrutiny of token women’s performance allows token men to showcase and exploit their skills (Williams, 1995a; Yoder and Kahn, 2003). In her interviews of nearly 100 men working in traditionally female jobs, Williams (1995b) found that token men’s achievements were often highlighted, and that their mistakes were rarely attributed to their gender. As a result, these men received preferential treatment in hiring decisions and greater incentives to remain in their jobs, as they were more often channeled into specialties with higher chances of upward mobility, or simply directly promoted.

This research suggests that evaluations of men are not subject to negative stereotype-based expectations, even in female-dominated occupations. Rather, it is argued that men’s perceived competence benefits from deeply embedded gendered beliefs within organizations, whereby stereotypically masculine qualities are equated with success, and stereotypically feminine qualities are devalued (Williams, 1995b). According to this view, the historical preference for agency over communality in the workplace overrides the effects of numerical dominance. As a result, men always have an advantage over women, even in female-dominated occupations (Williams, 1995b; Evans, 1997; Mahony et al., 2004).

Perhaps the most well-known (and well-documented) consequence of the “glass escalator” is the increased upward mobility of men in traditionally female fields. Several studies have provided support for this phenomenon. For example, token men have been found to receive more promotion recommendations and salary increases than token women and non-token men (Floge and Merrill, 1986; Heikes, 1991; Yoder, 1994; Barnett et al., 2000). Similarly, longitudinal studies using archival data found that men are more likely than women to move into managerial positions as the proportion of women in an occupation increases (Maume, 1999; Hultin, 2003). Other studies have shown that, in female-dominated occupations, men (White men in particular) are more likely than women to be promoted and to receive organizational benefits that enhance career opportunities (Baron and Newman, 1990; Cameron, 2001; Cognard-Black, 2004; Wingfield, 2009; Smith, 2012; Woodhams et al., 2015).

Beyond promotion opportunities, there is other evidence of male advantage in female-dominated contexts. In an experimental study, Fuegen and Biernat (2002) found that token men were positively evaluated by their teammates. Further, qualitative studies suggest that men are often aware of their advantage, describing how being a man in a female-dominated field can help to secure jobs and often leads to greater job stability (Yang et al., 2004; Lupton, 2006). Research in early education, a domain that is perceived to be highly female-typed (Croft et al., 2015; Tellhed et al., 2017) has shown that even when controlling for actual performance and job experience, male teachers are more likely to be hired over female teachers (McKenna and Johnson, 1981). Moreover, a meta-analysis of early educators (Borman and Dowling, 2008) found significantly less attrition among male than female teachers, providing indirect support for the idea men in female fields may be given more incentives than women to remain in their jobs. Supporting the idea that these positive outcomes stem from masculine organizational cultures, it has been argued that recent efforts to “professionalize” early education perpetuate beliefs linking competence to stereotypically masculine characteristics, even in this highly female-dominated field (Mahony et al., 2004). As a result, male teachers are often advantaged in selection, performance evaluations, and subsequent promotion opportunities. Interestingly, the “leaky pipeline” for men in female-dominated fields described above seems to disappear when men occupy higher-status positions (Torre, 2018).

Along with increasing men’s chances of selection, upward mobility, and salaries, gender stereotypes may benefit men in female-typed occupations in less tangible ways. Compared to women in male-typed roles and occupations, men in female-typed domains often describe higher perceptions of workplace support and lower perceptions of workplace mistreatment (Ott, 1989; Taylor, 2010). It has been argued that this may be due to differential task requirements and expectations for men and women in female-typed occupations (Williams, 1995b; Yang et al., 2004; Snyder and Green, 2008). For example, men in traditionally female jobs are not expected to engage in emotional labor to the same extent as their female peers (Cottingham et al., 2015). Thus, the perceived mismatch between men and the communal aspects of female-typed jobs may protect them from some of the psychological stressors (e.g., emotional demands, abusive emotional treatment) that are oftentimes inherent to care-related work (Hochschild, 1983; Evans, 1997).

Contrary to CMDs, the research summarized above suggests that men may in fact benefit from gender stereotypes, even when the setting is heavily female-dominated. However, the conclusions that can be drawn from this work come with their own set of limitations. Some of the findings outlined here have been called into question by other researchers. For example, several large-scale studies have found little evidence that men in female-dominated fields benefit from their token status (e.g., Budig, 2002) or that they are promoted more frequently than women (e.g., Snyder and Green, 2008; Price-Glynn and Rakovski, 2012).

Further, even if male-advantage exists for certain female-typed roles and settings, the specific processes underlying such advantage remain largely unclear. One possibility is that men’s opportunities are indeed enhanced (and women’s opportunities limited) by an overarching organizational culture that places more value on agency over communality. It is also possible that men’s advantage is a consequence of stereotypes of male competence being more impervious to contextual forces than female stereotypes. However, this observed male advantage may also reflect a different process altogether. Specifically, it is possible that some aspects of the “glass escalator” phenomenon could itself be explained via CMDs. In particular, enhanced promotion opportunities may be fueled by stereotype-based perceptions of incongruity between men and lower-level positions, and perceptions of congruity between men and higher-level positions, even in settings that have been traditionally dominated by women. Furthermore, within female-typed settings, the specific occupations to which men (but not women) are often channeled tend to be ones that are more aligned with masculine stereotypes (Yang et al., 2004; Levanon and Grusky, 2016). It may be that these positions offer more expedited paths to promotion. In this case, the perceived mismatch predicted by CMDs may in fact be symmetrical for men and women, but the consequences of such perceptions may not be equivalent.

On the other hand, stereotypes may play no role whatsoever in these effects. It is also possible that men’s advantage in female-typed occupations is merely a product of ingroup favoritism, fostered by the higher proportion of men in evaluative and decision-making positions. Thus, without more carefully controlled experimental studies that could directly explore the mechanisms underlying these effects, it is difficult to elucidate the causes of male-advantage and to determine the role, if any, of gender stereotypes and congruity perceptions in this process.

## Reexamining Congruity Models: Do Men Face Discrimination in Female-Dominated Occupations?

The previous section described two broad lines of research examining the evaluations of men in counterstereotypical domains, each reaching a different conclusion. While the first of body of work supports the predictions made by CMDs by presenting evidence of anti-male bias in female-typed settings, the second challenges these predictions and suggests that men may in fact have an advantage over women in traditionally female fields. However, both lines of research agree on one point: gender bias in evaluations exists. Notably absent from this review (and the literature in general) are studies that have failed to find evidence of bias – that is, research that has yielded no differences in evaluations of women and men in gender-incongruent settings. Such studies are likely to be greatly underrepresented both in previous analyses and in the present review due to a long history of publication pressures favoring significant over null results (Song et al., 2000; Dwan et al., 2008). This may have led to a general overestimation of the effects of gender stereotypes on the evaluations of both women and men. However, publication bias may be particularly problematic in the case of men in traditionally female roles and occupations, given the generally sparse amount of research in this area. For example, it is possible that the scarcity of published work is not the result of an actual lack of empirical studies, but of a “file drawer problem” (Iyengar and Greenhouse, 1988). That is, researchers may have indeed examined evaluations of men in female-typed domains but found no evidence of bias. Recent shifts in publication guidelines and increased openness to publishing null findings may therefore have the potential to improve our understanding of the power and scope of CMDs and to test their implications more rigorously.

Despite these shortcomings, the growing number of published studies examining evaluations of both women and men in gender-balanced and female-dominated fields, and the recent development of statistical tools to test and correct for publication bias (e.g., Duval and Tweedie, 2000) has greatly strengthened the conclusions that can be drawn from meta-analytical efforts. Interestingly, the two most recent meta-analyses comparing evaluations of women and men in female-typed occupations have reached a different conclusion from those of the dominant theoretical perspectives in the literature. In contrast with *both* congruity model and male-advantage predictions, these recent analyses suggest that men are neither disfavored nor favored in female-typed jobs. Specifically, Koch et al., 2015 meta-analysis found strong evidence of anti-female bias in male-typed roles and occupations but did not reveal symmetrical effects for men in female-typed positions. For men, the overall gender-based bias in female-dominated jobs was non-significant. Similarly, the 2014 meta-analysis by Paustian-Underdahl et al. (2014) found that, on average, evaluations of male leaders did not differ significantly from evaluations of female leaders in female-typed organizations.

Thus, the most recent and comprehensive analyses suggest that gender-based bias is not fully symmetrical and that different processes might be at play for evaluations of women and men in gender-incongruent roles and occupations. Nevertheless, there is reason to believe that this conclusion too should be interpreted with some caution. Though the number of studies examining female fields included in recent meta-analyses has certainly increased from earlier endeavors (e.g., Eagly et al., 1995; Davison and Burke, 2000), the imbalance in the number of studies focusing on female versus male fields remains substantial. In addition, the variety of female-typed fields included in these analyses is rather limited, often being restricted to one or two settings (particularly education). Additionally, a moderate portion of the research conducted in traditionally female domains (much of which was included in this review) is qualitative, which precludes it from being included in most meta-analyses. Thus, although these meta-analyses likely constitute the most systematic and reliable test of the symmetry hypothesis of CMDs yet, their results nonetheless do not reflect the full body of empirical findings on this topic.

In sum, the literature to date yields conflicting findings regarding whether gender discrimination truly is symmetrical, as is proposed by CMDs. Identifying where these discrepancies lie appears to be an important first step toward shaping the direction of future research that can further refine our theoretical understanding of gender bias. Indeed, the inconsistencies revealed in this review suggest that CMDs would greatly benefit from systematic research that directly tests its premises for women and men alike.

### Reexamining Congruity Models: The Domestic Sphere

Although women spend less time on domestic work than they did in the past, they continue to contribute significantly more than men to childcare and most household tasks, a disparity that negatively affects women’s career progress (Schoppe-Sullivan et al., 2013; Pew Research Center, 2014; Sullivan et al., 2018). Recently, several researchers have argued that to achieve true gender equality, a more balanced distribution of domestic labor is just as important as women’s full participation in the workplace. To this end, an emerging body of research in social psychology has begun to examine the reasons behind men’s lack of engagement in traditionally female work, including domestic labor (e.g., Vandello et al., 2013; Croft et al., 2015; Gutsell and Remedios, 2016; Meeussen et al., 2016; Tellhed et al., 2017).

Although CMDs have primarily been used to explain gender-based discrimination in the workplace, these models also have the potential to offer important insight into the processes involved in men’s lack of participation in domestic labor. Indeed, the domestic sphere may in fact be the domain in which we are most likely to observe the anti-male bias that is predicted by these models. Thus, broadening the purview of CMDs to include unpaid domestic work may in fact prove to be essential to decisively testing their theoretical predictions.

Just as paid labor has been historically dominated by men, unpaid domestic labor has traditionally been the domain of women. It has been argued that very few paid occupations are as female dominated as household work (Cohen, 2004). As a result, people continue to hold strong associations between women and the domestic sphere (Miller and Borgida, 2016), as well as the roles and behaviors that domestic labor entails (e.g., parenting, caretaking; Park et al., 2010). I contend that being a successful homemaker is likely to be perceived as requiring significantly more communality than agency. If so, the domestic sphere would appear to be the most direct analog to the male-typed roles and occupations in which CMDs have so frequently been tested. As such, unpaid domestic work may be the most appropriate setting for testing the symmetry of CMDs – the same perceived incongruity that gives rise to presumptions of lesser female competence in traditionally male occupations should also lead to the belief that men are not equipped to perform well in the household. The strong female-typing of domestic labor may render it one of the few domains in which women should have a clear advantage and be evaluated as significantly more competent than men.

The extant literature does not offer much evidence regarding whether men are indeed presumed to be less competent in the domestic sphere, nor whether these perceptions (to the degree that they exist) lead to discrimination against men in this domain. Perhaps the same reasons behind the dearth of research on evaluations of men and women in female-dominated occupations are also responsible for the scarcity of empirical studies on people’s perceptions of male and female homemakers’ ability. Because of its low status and unrecognized economic value, domestic labor is often assumed to be undesirable, especially for men. Indeed, the core components of this work (e.g., the care of children and the elderly, household chores) receive little to no monetary reward (United Nations Women, 2018). It is perhaps unsurprising, then, that the lack of male participation in household endeavors has rarely been interpreted as a possible product of gender-based discrimination. After all, domestic work has not been greatly sought after by men.

Nevertheless, the question of whether gender stereotypes about the domestic sphere play a role in men’s underrepresentation in domestic labor is an important one. Men’s reported interest in sharing domestic work is larger than ever before, and women are increasingly demanding more involvement from their male partners (Pew Research Center, 2013; Livingston, 2014; Dotti Sani and Treas, 2016). Thus, delving into the reasons behind men’s persistent lack of involvement – despite their increasing expressions of interest – is both timely and practically relevant. Examining whether there is evidence of anti-male bias in the domestic sphere also has important theoretical implications for CMDs. If men are thought to be less competent in childrearing and household tasks, and if these perceptions lead to the exclusion of men from domestic labor, this would provide strong evidence in support of the symmetry predicted by CMDs. Further, such a finding would suggest that incongruity perceptions may represent an additional barrier to the equal participation of women and men in domestic labor.

Though limited, there is some evidence to suggest that the domestic sphere is an area in which women’s competence is assumed. Arguing for a dynamic view of gender stereotypes, Mendoza-Denton et al. (2008) suggested that perceptions of male and female competence actually reverse in the context of domestic work. Specifically, when the context is framed as domestic rather than employment, women are described as more agentic than men (Mendoza-Denton et al., 2008). This is consistent with other research showing that, unlike in paid labor, women often hold a position of authority in the household, directly managing and planning most domestic tasks. This role includes taking charge of the majority of physical and psychological labor, as well as making most of the decisions related to childcare, family healthcare, household purchases, and beyond (Pew Research Center, 2008, 2015; Williams and Chen, 2014; Ciciolla and Luthar, 2019).

There is also some evidence that men’s domestic competence is viewed negatively, particularly in the case of childrearing. Poll data show that only 1% of people believe that fathers do a better job caring for a baby (vs. 53% favoring mothers). Further, among those who believe children are better off having at least one parent at home, only 2% think that parent should be the father (Pew Research Center, 2016, 2017a). Other research shows that, when asked to choose who should have custody of a child, most people (including judges) favor mothers over fathers, even when controlling for the characteristics of the parents (Miller, 2018). Beliefs about men’s lesser childrearing competence are also reflected in the media. For example, portrayals of “inept fathers” in advertising were recently found to be pervasive enough to prompt regulation in the United Kingdom (Advertising Standards Authority, 2018).

Family psychologists have described a phenomenon called “maternal gatekeeping” that further supports the idea that men’s competence may be put into question within domestic contexts. Maternal gatekeeping refers to the belief (observed mostly among mothers in the context of childrearing) that men are not as qualified as women to handle important domestic tasks and should therefore be prevented from performing them (Allen and Hawkins, 1999; Schoppe-Sullivan et al., 2004). While the literature offers a comprehensive description of the behaviors involved in maternal gatekeeping, the exact mechanisms underlying such behaviors remain unclear. For example, some research suggests that maternal gatekeeping hinders men’s childrearing abilities by limiting fathers’ involvement with their children (e.g., Allen and Hawkins, 1999; Altenburger et al., 2018). Other research argues for the reverse causal direction, suggesting that maternal gatekeeping is a protective strategy used to shield children from already incompetent fathers (e.g., Waller and Swisher, 2006; Austin et al., 2013). Further research is necessary to determine the extent to which maternal gatekeeping actually occurs and whether it is a product of stereotype-based expectations. If the psychological processes behind maternal gatekeeping indeed arise from culturally shared beliefs regarding what men and women are like and what it takes to be a good homemaker, then this phenomenon may provide support for the symmetry of CMDs.

In addition, the perceived mismatch between male stereotypes and the communal requirements for success in domestic labor might also impact men’s own perceptions of competence in this domain. A large body of work has demonstrated that women tend to internalize gender stereotypes and come to believe that they are less efficacious than men in traditionally male fields (e.g., Eccles, 1994; Correll, 2004). Decreased self-efficacy has been associated with a lower sense of belonging among women than men in traditionally male settings (Good et al., 2012), as well as lower motivation to participate and engage in these areas (Cheryan et al., 2015). Similarly, men too may internalize stereotype-based expectations about their own lack of proficiency in child-rearing and household chores and conclude that they do not have what it takes to perform well in domestic roles, an idea that has found some support in qualitative research (e.g., Miller, 2011; Ives, 2014). These beliefs, in turn, may lead men to avoid parental responsibilities and to exclude themselves from domestic labor altogether, deferring to women as the domestic “experts.”

It is important to note that, like paid labor, unpaid domestic labor is itself divided into roles and tasks that are likely to be differentially gender-typed. Though women spend significantly more time on domestic work than men (even when both partners are employed), there is variation among different forms of domestic work. For example, women report spending more time cooking and cleaning than men do, but men report spending more time on garden maintenance and repairs than women do (American Time Use Survey, 2017). The relative distribution of men and women in these different roles may influence their perceived gender-type and future research examining evaluations of men’s (vs. women’s) perceived domestic competence should consider these distinctions.

Certainly, the perceived mismatch between male stereotypes and domestic labor is not the sole explanation for men’s lack of domestic engagement. Other psychological mechanisms, including motivational processes, are likely to contribute to the belief that men are not good homemakers and that they should not participate in domestic labor. For example, both men’s and women’s motivation to uphold the status-quo has been associated with the endorsement of gender stereotypes (Glick and Fiske, 2001; Jost and Kay, 2005) and therefore may also explain men’s lower involvement in the household. Furthermore, even though the imbalance in domestic work can have negative consequences for women’s career advancement, some women may be particularly motivated to maintain ownership over the domestic sphere. As a domain in which female competence is likely acknowledged and unquestioned, domestic labor may be one of the few areas that is primarily reserved for women. Indeed, research suggests that women derive a sense of control from the relative power that gender stereotypes garner them in the household (Williams and Chen, 2014). Moreover, many women may have a strong sense of pride deriving from their (real or perceived) domestic superiority and may strongly identify as mothers and homemakers. For these women, greater male involvement in child-rearing and domestic work may be perceived as a threat to their status within the household and to their identity.

In sum, the dearth of research examining perceptions of domestic competence does not yet allow for a rigorous test of CMDs predictions in this domain. Nevertheless, there is indirect evidence to suggest that, just as incongruity beliefs give rise to the expectation that women will be less competent in male-typed jobs, men too may be deemed less competent in the female-typed household. It is possible, then, that the failure to find clear support for the symmetry of CMDs lies, in part, in the general omission of the domestic sphere as a relevant setting in which to test its assumptions. Examining whether symmetrical incongruity beliefs exist about men in the household, and whether these beliefs lead to perceptions of female superiority and male incompetence, would provide a more thorough and decisive test of CMDs. Importantly, this research would allow us to better understand the processes underlying men’s continued lack of domestic participation, a phenomenon that continues to hinder the attainment of gender equality.

### Prescriptive Gender Stereotypes and the Evaluation of Men in Female-Typed Settings

The main goal of this paper was to critically examine congruity models of gender discrimination in light of the extant literature on evaluations of men in female-typed fields. To this end, I focused on descriptive gender stereotypes and their consequences for the competence perceptions of men in traditionally female roles and occupations. However, as mentioned earlier, gender discrimination is not only the product of a mismatch between the perceived requirements of a position and descriptive gender stereotypes (“what men and women are like”); it also results from violations to prescriptive gender stereotypes (“what men and women should be like”). Specifically, prescriptive gender stereotypes may lead to discrimination through social penalties and backlash. Much research has shown that women who are thought to violate these stereotypes (e.g., by behaving in a dominant way or displaying competence in a male-typed roles) are disliked and seen as less hireable (Rudman et al., 2012; Williams and Tiedens, 2016). Though the particular social penalties incurred by men who choose to do “women’s work,” be it paid female-typed labor or unpaid domestic labor, were beyond the scope of this paper, they are surely crucial to fully understanding men’s lack of participation in traditionally female domains. Examining whether these penalties and their downstream consequences are equivalent for male and female gender transgressors is an important question that this paper did not address.

Several authors have argued that prescriptive gender stereotypes and femininity injunctions for men play an important role in men’s underrepresentation in communal roles and occupations (Thompson et al., 1985; Croft et al., 2015; Meeussen et al., 2016; Tellhed et al., 2017). A growing body of research has shown that, like women, men too are punished for violating gender norms by behaving in gender-incongruent ways. For example, men who demonstrate proficiency in female-dominated occupations are seen as weak and undeserving of respect (Heilman and Wallen, 2010) and often encounter social backlash (Rudman and Fairchild, 2004). Similarly, modest and self-effacing men are frequently derogated by others (Rudman, 1998; Moss-Racusin et al., 2010). Some have argued that the penalties for gender norm violations are not equivalent for men and women, and that men actually incur *greater* social costs due to stricter masculinity prescriptions (Pleck, 1995; Vandello and Bosson, 2013). These increased penalties may stem from the fact that stereotypes prescribing agentic behavior for men are often compounded by the strong association between feminine men and homosexuality (Kite and Deaux, 1987), an association that appears to be less strong in the case of masculine women. It has been argued that the fear of being perceived as homosexual may be enough to lead many men to actively avoid communal behaviors and activities (Bosson et al., 2013).

Although the penalties for violating gender norms are mostly informal (e.g., dislike, derogation, avoidance), they may still result in discrimination against men in communal roles and occupations by promoting the exclusion – and self-exclusion – of men from these domains. Thus, even if men and women are selected at equal rates, or if they climb the organizational ladder more quickly, men may still be deterred from pursuing a career in female-typed areas because of the harsh social penalties that such a decision might entail. Field research supports this idea, describing how men in traditionally female occupations often express fear about how they will be perceived by others. For example, male nurses and early childhood educators report being afraid of having their masculinity questioned and, in particular, of being seen as socially and sexually deviant (Williams, 1995a; Cameron, 2001; Harding, 2007). These fears are likely to play an important role in men’s job pursuits and aspirations, including their decision to enter and remain in female-typed occupations.

Prescriptive gender stereotypes can also result in penalties for men engaging in unpaid domestic labor. Research has shown that actively taking time off work to fulfill family responsibilities leads to negative consequences not only for women, but for men as well (Wayne and Cordeiro, 2003; Butler and Skattebo, 2004; Coltrane et al., 2013; Rudman and Mescher, 2013). For example, men who spend a significant amount of time dedicated to their family report experiencing more workplace harassment and mistreatment than women in similar caregiving roles (Berdahl and Moon, 2013). This may be due to the fact that employees seeking family-related work flexibility are often described in feminine terms, a perception that results in social penalties for men, but not women who actively pursue greater domestic participation (Vandello et al., 2013).

In sum, prescriptive stereotypes may contribute to men’s lack of participation in female-typed roles and occupations by fostering a hostile environment for men who choose to engage in this type of labor. Future research should explore whether the processes that lead to social penalties are similar for men and women, and whether the consequences for prescriptive violations are comparable. It is possible, for example, that the strong association between male stereotypes and the provider role might shield norm-violating men from the economic costs of backlash (e.g., decreased hireability and promotion), but that men are more likely than women to lose their social standing as a result of their transgression, given the lower status assigned to female roles and behaviors.

## Conclusion

The present review examined the literature on evaluations of men in female-typed settings with the goal of elucidating whether discrimination processes for men and women are truly symmetrical, as congruity models of discrimination predict. The results were mixed. While some research provides support for the idea that men, like women, are presumed to be less competent in gender-incongruent occupations, other research suggests that men may have an advantage over women in female-dominated occupations.

However, these findings do not necessarily imply that CMDs are only useful when explaining discrimination against women. Expanding the paradigms used to test CMDs to also include unpaid domestic work has the potential to deepen and refine our understanding of gender discrimination, as well as to provide further support for the psychological processes underlying these models. Future research should explore whether the mismatch between male stereotypes and domestic stereotypes give rise to perceptions that men are less competent in the domestic sphere. Doing so may help to identify important predictors of men’s lack of engagement and participation in the household and can shed light on potential pathways to balance the distribution of women and men, *both* in the workplace and the household.

## Author Contributions

The author confirms being the sole contributor of this work and has approved it for publication.

## Conflict of Interest Statement

The author declares that the research was conducted in the absence of any commercial or financial relationships that could be construed as a potential conflict of interest.
